# Changes in Clinical Characteristics of Community-Acquired Acute Pyelonephritis and Antimicrobial Resistance of Uropathogenic *Escherichia coli* in South Korea in the Past Decade

**DOI:** 10.3390/antibiotics9090617

**Published:** 2020-09-18

**Authors:** Ki Tae Kwon, Bongyoung Kim, Seong-yeol Ryu, Seong-Heon Wie, Jieun Kim, Hyun-uk Jo, Se Yoon Park, Kyung-Wook Hong, Hye In Kim, Hyun ah Kim, Mi-Hee Kim, Mi Hyun Bae, Yong-Hak Sohn, Jieun Kim, Yangsoon Lee, Hyunjoo Pai

**Affiliations:** 1 Department of Internal Medicine, School of Medicine, Kyungpook National University, Daegu 41566, Korea; ktkwon@knu.ac.kr; 2 Department of Internal Medicine, College of Medicine, Hanyang University, Seoul 04763, Korea; quidam76@hanyang.ac.kr; 3 Department of Internal Medicine, Keimyung University Dongsan Medical Center, Daegu 41931, Korea; 121rsy@dsmc.or.kr (S.-y.R.); hyunah1118@dsmc.or.kr (H.a.K.); 4 Division of Infectious Diseases, Department of Internal Medicine, St. Vincent Hospital, College of Medicine, The Catholic University of Korea, Seoul 06591, Korea; wiesh@catholic.ac.kr (S.-H.W.); miheekim3528@gmail.com (M.-H.K.); 5 Department of Urology, School of Medicine, Eulji University, Daejeon 34824, Korea; hujo@eulji.ac.kr; 6 Division of Infectious Diseases, Department of Internal Medicine, Soonchunhyang University Seoul Hospital, Soonchunhyang University College of Medicine, Seoul 04401, Korea; sypark@schmc.ac.kr; 7 Division of Infectious Diseases, Department of Internal Medicine, Gyeongsang National University Hospital, Gyeongsang National University School of Medicine, Jinju 52727, Korea; resina78@gnuh.co.kr; 8 Department of Internal Meidcine, Daegu Fatima Hospital, Daegu 41199, Korea; cheonlang1@hanmail.net; 9 Department of Laboratory Medicine, College of Medicine, Hanyang University, Seoul 04763, Korea; mhbae@hanyang.ac.kr (M.H.B.); yangsoon@hanyang.ac.kr (Y.L.); 10 Seegene Medical Foundation, Seoul 04805, Korea; medsohn@naver.com; 11 Department of Laboratory Medicine, Soonchunhyang University Seoul Hospital, Soonchunhyang University College of Medicine, Seoul 04401, Korea; jkim1220@schmc.ac.kr

**Keywords:** pyelonephritis, urinary tract infection, antimicrobial resistance, Korea

## Abstract

This study examined changes in the clinical characteristics of community-acquired acute pyelonephritis (CA-APN) in South Korea between the period 2010–2011 and 2017–2018. We recruited all CA-APN patients aged ≥19 years who visited eight hospitals in South Korea from September 2017 to August 2018, prospectively. Data collected were compared with those from the previous study in 2010–2012, with the same design and participation from 11 hospitals. A total of 617 patients were enrolled and compared to 818 patients’ data collected in 2010–2011. *Escherichia coli* was the most common causative pathogen of CA-APN in both periods (87.3% vs. 86.5%, *p* = 0.680). *E. coli* isolates showed significantly higher antimicrobial resistance against fluoroquinolone (32.0% vs. 21.6%, *p* < 0.001), cefotaxime (33.6% vs. 8.3%, *p* < 0.001), and trimethoprim/sulfamethoxazole (37.5% vs. 29.2%, *p* = 0.013) in 2017–2018 than in 2010–2011. Total duration of antibiotic treatment increased from 16.55 ± 9.68 days in 2010–2011 to 19.12 ± 9.90 days in 2017–2018 (*p* < 0.001); the duration of carbapenem usage increased from 0.59 ± 2.87 days in 2010–2011 to 1.79 ± 4.89 days in 2010–2011 (*p* < 0.001). The median hospitalization was higher for patients in 2017–2018 than in 2010–2011 (9 vs. 7 days, *p* < 0.001). In conclusion, antimicrobial resistance of *E. coli* to almost all antibiotic classes, especially third generation cephalosporin, increased significantly in CA-APN in South Korea. Consequently, total duration of antibiotic treatment, including carbapenem usage, increased.

## 1. Introduction

Antimicrobial resistance is a global crisis for humans and threatens progress in health and achievement of the Sustainable Development Goals [1]. Once a pathogen acquires resistance, it affects negatively the prognosis of patients such as an increase in mortality, length of hospital stay, and medical costs [2]. In particular, multidrug Gram-negative bacteria, including carbapenem-resistant *Enterobacteriales* and carbapenem-resistant *Acinetobacter baumannii*. were considered as “urgent threat” pathogens by the Centers for Disease Control and Prevention in 2019 and require special precautions [3].

Acute pyelonephritis (APN) is one of the most common infectious diseases mainly caused by Gram-negative bacteria in the community. It usually responds well to antimicrobial agents, however, increasing the proportion of antimicrobial-resistant pathogens makes its treatment challenging [4]. Unfortunately, *E. coli,* which comprise more than 90% of cause of urinary tract infection (UTI), has become more resistant to multiple antibiotics commonly used for the treatment of APN in recent years [5]. As observed in other parts of the world, the increasing resistance of *Enterobactericiales*, including *E. coli*, against several antibiotics were observed in South Korea [6]. In discussing *E. coli* isolates from uncomplicated UTIs, although the resistance rate against carbapenem still remains low, the increase in fluoroquinolone (FQ)-resistance was significant in the last decade [7,8].

Not only increase in antimicrobial resistance, but also change in population structure and healthcare system may affect the characteristics of APN. As known, South Korea has been experiencing rapid aging and is expected to become one of the most aged countries in the world [9]. Furthermore, entry barriers to the hospitals have decreased due to the improvement of the social welfare system and the rapid expansion of private medical insurance in the last decade [10]. Accordingly, we could find that the epidemiologic characteristics of APN, such as the incidence and medical cost, as well as antibiotic prescription patterns for the treatment of APN, have changed in South Korea [10,11].

However, the change in clinical and microbiological characteristics of APN has not been assessed properly yet. The aim of the present study is to identify the change in clinical and microbiological characteristics of community-acquired APN (CA-APN) in South Korea.

## 2. Material and Methods

### 2.1. Study Setting

**Figure 1:**
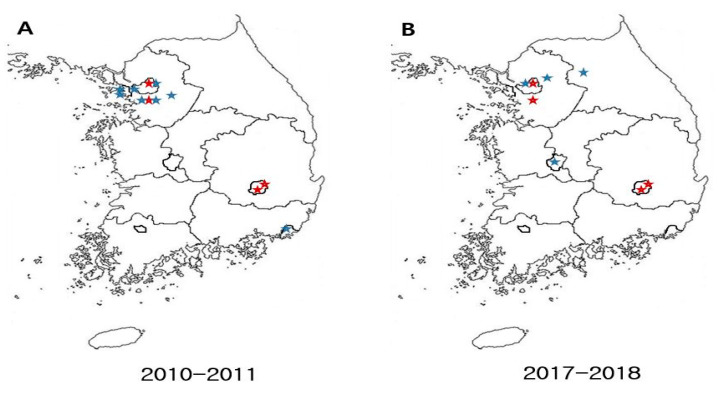
Geographic distribution of hospitals that participated in the study. Red stars indicate the hospitals that participated in both cohorts. (**A**) 11 hospitals for the study in 2010–2011, (**B**) 8 hospitals for the study in 2017–2018.

A prospective cohort study was carried out in 8 hospitals in South Korea, from 1 Sep 2017 to 31 Aug 2018. The participating hospitals had 580–915 beds and were located through the Korean peninsula (2 in Seoul, 2 in Gyeonggi-do, 1 in Daejeon, 2 in Daegu, and 1 in Gangwon-do) and 7 of 8 were university-affiliated hospitals: Keimyung University Dongsan Hospital (915 beds); Daegu Fatima Hospital (733 beds); Eulji University Hopstial (859 beds); St. Vincent’s Hospital (902 beds); Soonchunhyang University Seoul Hospital (717 beds); Hanyang University Guri Hospital (580 beds); Hanyang University Seoul Hospital (846 beds); Chuncheon Sacred Heart Hospital (829 beds). The collected data were compared with those from the previous study with same design in 2010–2011, in which 11 hospitals in South Korea participated (4 of 11 participated in both cohort) [12] (Figure 1).

### 2.2. Patient Population

We recruited all adult APN patients (age ≥ 19 years) who were hospitalized to the participating hospitals. Cases were enrolled at admission by infectious diseases or urology specialists of each hospital. The inclusion criteria were: (i) presence of fever (body temperature ≥ 37.8 °C), (ii) pyuria (≥5–9 white blood cells per high power field (WBC/HPF)), and (iii) clinical symptoms or signs relevant to APN judged by infectious diseases or urology specialists in each hospital. Patients diagnosed with APN more than 48 h after admission, those transferred from other hospitals during treatment of APN, those with other reasons for fever and pyuria, and those with insufficient data were excluded. Each patient was included for the first episode during the study period.

### 2.3. Microbiological Data and Definitions

For analyses of causative pathogens and their antibiotic susceptibilities, blood or urine specimens collected at the time of admission were processed at laboratories of each hospital. Etiologic agents were determined when organisms at ≥10^5^ Colony-forming unit (CFU)/mL were identified in urine culture, and/or pathogens that were not considered as contaminants (e.g., coagulase-negative *Staphylococcus, Bacillus* spp., viridans group *Streptococci, Corynebacterium* spp., *Propionibacterium* spp. and *Micrococcus* spp.) were isolated from blood culture [13,14,15]. Identification of bacterial species and their susceptibilities to antibiotics were determined by means of a semi-automated system (VITEK, bioMèrieux, Hazelwood, MO, USA, or Microscan, Dade Behring, West Sacramento, CA, USA). The breakpoints of each compound were defined in reference to the Clinical and Laboratory Standards Institute [16], and R (resistance) or I (intermediate) were defined as resistance. We defined extended-spectrum cephalosporins (ESC) as 3rd generation cephalosporins or 4th generation cephalosporins.

### 2.4. Clinical Data and Definitions

We collected data about (i) demographic features (age and sex), (ii) risk factor variables, (iii) initial clinical characteristics, (iv) antibiotic prescription, and (v) clinical outcomes.

Risk factor variables consisted of underlying co-morbidities, underlying structural or functional abnormalities of the urinary tract [17,18], and additional past histories associated with extended-spectrum beta-lactamase (ESBL) acquisition [12]. Underlying co-morbidities included components of Charlson’s comorbidity index [19], pregnancy, menopause, and bedridden state. Underlying urinary tract conditions were: indwelling urinary catheter, intermittent catheterization, benign prostatic hyperplasia (BPH), neurogenic bladder, urolithiasis, urinary retention, vesicoureteral reflux, vaginal wall prolapse, polycystic kidney, and renal tumor. Additional past histories associated with ESBL acquisition were: history of admission and antibiotic usage during 1 year prior to inclusion, history of UTI, use of chemotherapeutic agents and immunosuppressants, history of urinary catheterization during 1 month prior to inclusion, and history of urinary tract operation during 3 months prior to inclusion.

Initial clinical features included Pitt’s bacteremia score [20], UTI symptoms such as dysuria, urgency, frequency, and nocturia, back pain, vomiting/diarrhea, hematuria (≥5–9 red blood cells per high power field (RBC/HPF)), azotemia (serum blood urea nitrogen (BUN) ≥20 mg/dL and/or serum creatinine ≥1.4 mg/dL), and presence of bacteremia at the time of admission.

Classes, administration route, and duration of antibiotics prescribed for APN treatment were recorded. The initial antibiotic regimens were considered to be concordant if it included at least one antibiotic active against the causative organisms on in vitro susceptibility testing and if the dose and route of administration conformed to current medical standards [21].

For assessment of outcomes, we evaluated clinical failure rate, hospitalization days, and febrile days. Clinical failure was defined as recurrence of UTI symptoms within 7–14 days after the completion of therapy or death. Patients who did not have a follow-up visit were excluded from the analysis.

### 2.5. Statistical Analyses

All statistical analyses were conducted using SPSS version 24.0 for Windows (IBM Corp., Armonk, NY, USA). Categorical variables were analyzed using the Chi-square test or Fisher’s extract test, as appropriate. Continuous variables were analyzed by the Mann–Whitney U test or independent *t*-tests. We also performed a propensity-score matching analysis with a matching weight of 1:1 to reduce the effects of confounding. Age and Charlson’s comorbidity index were included for the modeling and non-matched cases were discarded for the subanalysis. A two-tailed *p*-value of <0.05 was considered statistically significant.

### 2.6. Ethical Statement

The study protocol was approved by the Institutional Review Board (IRB) of Hanyang University Seoul Hospital (IRB number: 2017-07-009) in addition to each hospital. The written informed consent from patients was obtained by researchers in each hospital.

## 3. Results

A total of 617 patients with CA-APN were enrolled during this study period. The clinical and microbiological data of those patients were compared with those of 818 patients with CA-APN in 2010–2011. Of 617 patients, 56.6% (349/617) were from four hospitals that participated in both periods. We also compared data of those patients with those of 472 from the same hospitals in 2010–2011.

### 3.1. Change in Causative Pathogens for CA-APN and Their Antibiotic Susceptibilities

Table 1 shows the distribution of causative pathogens for CA-APN. *E. coli* was overwhelmingly the most common pathogen in both studies comprising ≥85%. Other Enterobactericiales, such as *K. pneumoniae*, *Proteus* spp., *Enterobacter* spp., and *Citrobacter* spp., were also identified. There was no difference in the composition of uropathogens during both periods. Likewise, no difference in the composition of uropathogens was observed for the patients from the four hospitals that participated in both studies (Table S1).

Table 2 shows the antimicrobial susceptibilities of *E. coli* isolated from patients with community-acquired acute pyelonephritis (CA-APN). Compared with 2010–2011, antimicrobial resistance rates of uropathogenic *E. coli* against several antibiotics were significantly higher in 2017–2018: cefepime (8.1% vs. 31.7%, *p* < 0.001), cefotaxime (8.3% vs. 33.6%, *p* < 0.001), fluoroquinolone (FQ) (21.6% vs. 32.0%, *p* < 0.001), and trimethoprim/sulfamethoxazole (29.2% vs. 37.5%, *p* = 0.013). The resistance rate for amikacin, imipenem, meropenem, and piperacillin/tazobactam remained lower than 5% in both periods. A similar changing pattern was observed from 4 hospitals that participated in both periods (Table S2).

### 3.2. Comparison of Demographic Data and Risk Factors

Demographic data and risk factor variables are shown in Table 3. The mean age of patients with CA-APN was 56.66 ± 19.06 years in 2010–2011 and increased to 60.21 ± 18.73 years in 2017–2018 (*p* < 0.001). Females predominated (≥90%) in both study periods. As for the Charlson’s comorbidity index, the patients in 2017–2018 had higher score compared with those in 2010–2011 (0.81 ± 1.37 vs. 1.04 ± 1.40, *p* = 0.002). The proportion of patients with certain co-morbidities such as diabetes mellitus (26.4% vs. 31.8%, *p* = 0.026), malignancy (3.9% vs. 8.3%, *p* < 0.001), liver disease (3.2% vs. 5.8%, *p* = 0.014), dementia (3.9% vs. 6.2%, *p* = 0.05), and menopause (45.2% vs. 56.4%, *p* < 0.001) were significantly higher in 2017–2018 compared with 2010–2011. As for the underlying urinary tract conditions, a higher proportion of patients with indwelling urinary catheter (1.5% vs. 3.6%, *p* = 0.010), BPH (for male patients; 21.0% vs. 43.1%, *p* < 0.011), neurogenic bladder (0.9% vs. 4.1%, *p* < 0.001), and urolithiasis (1.8% vs. 3.6%, *p* = 0.040) were observed in 2017–2018 compared with 2010–2011. Similar differences were observed for the patients from the four hospitals that participated in both periods (Table S3).

### 3.3. Comparison of Clinical Characteristics

The overall clinical characteristics of patients with CA-APN in both periods are shown in Table 4. Fewer patients presented UTI symptoms in 2017–2018 compared with those in 2010–2011 (64.3% vs. 53.3%, *p* < 0.001). In comparison, more patients in 2017–2018 had azotemia (16.5% vs. 29%, *p <* 0.001) and bacteremia (32.0% vs. 43.6%, *p <* 0.001) compared with those in 2010–2011 at the time of admission.

In 2017–2018, the probability of receiving initial empirical antibiotics that were discordant to the antimicrobial susceptibility of the causative pathogens was higher than that in 2010–2011 (26.8% vs. 11.2%, *p* < 0.001). ESC, beta-lactam/beta-lactamase inhibitor, and carbapenem were used more frequently as initial empirical antibiotics in 2017–2018 while FQs were not chosen as frequently as before. Moreover, total duration of antibiotic therapy for the treatment of CA-APN increased significantly (16.55 ± 9.68 vs. 19.12 ± 9.90, *p* < 0.001). Notably, the duration of carbapenem usage increased from 0.59 ± 2.87 days in 2010–2011 to 1.79 ± 4.89 days in 2010–2011 (*p* < 0.001).

As for clinical outcomes, mortality and clinical failure rate decreased significantly. The mortality rate was 1.9% in 2010–2011 and decreased to 0.2% in 2017–2018 (*p* = 0.003); clinical failure rate was 5.3% in 2010–2011 and decreased to 2.2% in 2017–2018 (*p* = 0.003). In comparison, duration of hospitalization increased from 7 (interquartile range (IQR) 6–10) to 9 (IQR 7–13) days (*p* < 0.001). A similar changing pattern was observed for the patients from the four hospitals that participated in both periods (Table S4).

### 3.4. Comparison of Clinical and Microbiological Characteristics after Propensity-Score Matching

The overall clinical and microbiological characteristics of patients with CA-APN after propensity-score matching are shown in Table 5. In the matched analytic sample, 617 patients were included for each group, respectively. Alike the results from the analysis with unmatched samples, the antimicrobial susceptibilities of uropathogenic E. coli against several antibiotics, including ESC, FQ, and trimethoprim/sulfamethoxazole, were significantly higher in 2017–2018 compared with 2010–2011. In addition, in 2017–2018, the probability of receiving initial empirical antibiotics that were discordant to the antimicrobial susceptibility of the causative pathogens was higher and the total duration of antibiotic therapy for the treatment of CA-APN was longer than that in 2010–2011. The difference in clinical outcome variables between the periods 2010–2011 and 2017–2018 was similar to the results from the analysis with unmatched samples as well. In 2017–2018, the mortality and clinical failure rate were lower while the duration of hospitalization was longer than that in 2010–2011.

## 4. Discussion

As time goes by, the characteristics of disease change. According to recent studies about APN in South Korea using the claim database from 2010 to 2014, the annual incidence rate was on the increase year upon year and prescription of broad-spectrum antibiotics for the treatment of APN had increased [10,11]. In the present study, we identified the change in characteristics of CA-APN in South Korea from the clinical aspect.

One of the most significant findings is that antimicrobial-resistance had increased. The composition of uropathogens has not been changed for the last decade with *E. coli* still remaining overwhelmingly the most frequent causative pathogen for APN. Looking at *E. coli*, the antimicrobial resistance to almost all antibiotic classes including ESC and FQ, which have been accepted as first-line empirical antibiotics for APN, increased significantly [7]. Compared to the previous nationwide cohort study in which the proportion of ESC and FQ-resistant *E. coli* causing CA-APN comprised 7.6% and 21.3%, respectively, the current study revealed that the proportion of those pathogens increased to 33.6% and 32.0%, respectively [12,13]. A similar resistance rate for the community origin *E. coli* was observed in the national antimicrobial resistance surveillance system in Korea: the Korean global resistance surveillance system (Kor-GLASS) reported that the resistance rate of *E. coli* to ESC and FQ were approximately 30% and 40%, respectively, in 2017 [22].

The situation is similar in other countries. Increasing resistance of uropathogenic *E. coli* to multiple antibiotics has been reported worldwide. In the U.S., rates of ESBL-producing *E. coli* from UTI increased from 7.8% in 2010 to 18.3% in 2014 [23]. A longitudinal survey reported that ceftriaxone resistance among *E. coli* isolates increased from 9.6% in 2004 to 44.1% in 2016 in South America [24]. As for Asian countries, the proportion of ESBL-producing pathogens among *E. coli* isolates from UTI increased from 18.4% in 2010 to 28.1% in 2013 in Singapore; 15.8% in 2010 to 34% in Philippines; 25% in 2010 to 41.7% in 2013 in Hong Kong [25].

There are two possible mechanisms for the increase in the proportion of antimicrobial-resistant uropathogenic *E. coli*: vertical and horizontal transmission. As for the former one, the increase in the usage of broad-spectrum antibiotics might have contributed to the emergence of antimicrobial-resistant pathogens. According to a previous study in South Korea, there was significant correlation between nationwide FQ use and the increase in the proportion of FQ-resistant *E. coli* isolates. Similarly, nationwide cefotaxime use increased and was correlated with the increase in the proportion of cefotaxime-resistant *E. coli* isolates [26]. In addition, a stepwise increase in the consumption of broad-spectrum antibiotics, such as ESC and FQ, was observed at a Korean hospital over the last decade [27]. As for the aspect of horizontal transmission, the change in the major clonal type of uropathogenic *E. coli* strains might have affected the emergence of antimicrobial resistance. As observed in other countries, high-risk clones for multidrug-resistant *E. coli* strains, such as the ST131 clone, might have increased in South Korea. In the U.K., the proportion of ST131 clone among *E. coli* isolated from blood had increased from 2.9% in 2010 to 20.7% in 2010 [28]. Further studies about change in microbiologic characteristics in South Korea are necessary to clarify this issue.

We suggest that the increased antimicrobial resistance of *E. coli* might have resulted in the increase in the total duration of antibiotic treatment and hospitalization days. An increase in antimicrobial resistance rate to commonly prescribed antibiotic classes might have led to a more frequent mismatch between initial antimicrobial regimen and pathogens. Physicians should be alert to this phenomenon because an increasing consumption of antimicrobial agents causes the emergence of other antimicrobial-resistant pathogens [29]. Considering that carbapenem is one of the antibiotics used as a last resort to beat multidrug-resistant Gram-negative bacteria, increase in the proportion of carbapenem usage is a worrisome finding. In South Korea, the increase in the incidence of carbapenem resistant *Enterobactericeae* (CRE) carriage has become a serious problem [30]. To curb the vicious cycle, antimicrobial stewardship and prescription control of broad-spectrum antibiotics for the treatment of common but not serious infections, such as CA-APN, should be reinforced. Additionally, other antibiotics which could spare carbapenems for the treatment of CA-APN caused by ESBL-producing pathogens, such as piperacillin/tazobactam or gentamicin, should be considered for the treatment of less severe cases [31,32].

In the present study, patients were older and had higher Charlson’s comorbidity index score compared to those in 2010–2011. Despite this, the rate of current clinical failure or mortality is lower than it was 7 years ago. This might be associated with advances in medicine and the healthcare system in South Korea. In fact, the utilization of healthcare in South Korea is highest among the Organization for Economic Co-operation and Development (OECD) countries and is increasing year upon year [33]. Further studies are necessary to identify the cause of this phenomenon.

There are several potential limitations in our study. Firstly, this study was conducted mainly in large hospitals and only hospitalized patients were recruited. Therefore, enrolled patients in the study might have more underlying co-morbidities and the results cannot be generalized to the entire population. Secondly, there might be differences in the inclusion among the hospitals. Aside from fever and pyuria, clinical symptoms or signs relevant to APN were judged by physicians in each hospital. Thirdly, the number of co-morbidities, past histories and the amount of antibiotic consumption might be underestimated because the investigators in each hospital could not access the medical records of patients in other hospitals. Some of the information was obtained only through the interview with patients or caregivers. Fourthly, the change in the healthcare system and medical facilities was not assessed. Finally, the participating hospitals were not exactly the same compared to the previous study. In order to amend the limitation, we performed subgroup analyses for the hospitals that participated in both periods.

## 5. Conclusions

In conclusion, antimicrobial resistance of *E. coli* to almost all antibiotic classes, especially third generation cephalosporin, increased significantly in CA-APN in South Korea. Consequently, the total duration of antibiotic treatment and the proportion of carbapenem usage increased.

## Figures and Tables

**Table 1:** Comparison of the aetiologies of community-acquired acute pyelonephritis.

Pathogens	2010–2011	2017–2018	*p*
*Escherichia coli*	526 (86.5)	441 (87.3)	0.680
*Klebsiella pneumoniae*	24 (3.9)	27 (5.3)	0.270
*Proteus* spp.	9 (1.5)	7 (1.4)	0.892
*Enterobacter* spp.	5 (0.8)	5 (1.0)	0.763
*Citrobacter* spp.	1 (0.1)	5 (1.0)	0.098
Subtotal	565 (92.9)	485 (96.0)	0.025
*Pseudomonas aeruginosa*	5 (0.8)	5 (1.0)	0.763
*Acinetobacter baumannii*	2 (0.3)	0 (0)	0.504
*Enterococcus* spp.	10 (1.6)	4 (0.8)	0.203
*Staphylococcus aureus*	3 (0.5)	0 (0)	0.256
Others	23 (3.8)	11 (2.2)	0.120
Total	608 (100)	505 (100)	-

**Table 2:** Comparison of antibiotic susceptibilities of uropathogenic *E. coli* isolates.

	2010–2011 (*n =* 526)		2017–2018 (*n* = 441)		
Antibiotics	Sensitive (%)	Resistant (%)	Sensitive (%)	Resistant (%)	*p*
Amikacin (AMK)	516 (98.1)	10 (1.9)	440 (99.8)	1 (0.2)	0.014
Amoxicillin/Clavulanate (AMC)	307 (79.9)	77 (20.1)	267 (71.4)	107 (28.6)	0.006
Ampicillin (AMP)	204 (39.8)	308 (60.2)	128 (29.0)	313 (71.0)	<0.001
Ampicillin/Sulbactam (SAM)	84 (53.2)	74 (46.8)	55 (40.7)	80 (59.3)	0.034
Aztreonam (ATM)	430 (90.9)	43 (9.1)	304 (68.9)	137 (31.1)	<0.001
Cefazolin (CFZ)	232 (81.7)	52 (18.3)	237 (63.5)	136 (36.5)	<0.001
Cefepime (FEP)	474 (91.9)	42 (8.1)	301 (68.3)	140 (31.7)	<0.001
Cefotaxime (CTX)	472 (91.7)	43 (8.3)	292 (66.4)	148 (33.6)	<0.001
Cefoxitin (FOX)	363 (93.6)	25 (6.4)	409 (92.7)	32 (7.3)	0.644
Ceftazidime (CAZ)	476 (91.4)	45 (8.6)	302 (68.5)	139 (31.5)	<0.001
Fluoroquinolone (FQ)	409 (73.6)	113 (21.6)	300 (68.0)	141 (32.0)	<0.001
Gentamicin (GEN)	404 (77.4)	118 (22.6)	296 (67.1)	145 (32.9)	<0.001
Imipenem (IPM)	521 (99.8)	1 (0.2)	441 (100)	0 (0)	1.000
Meropenem (MEM)	395 (99.5)	2 (0.5)	135 (100)	0 (0)	1.000
Piperacillin (PIP)	161 (40.1)	240 (59.9)	33 (32.7)	68 (67.3)	0.168
Piperacillin/Tazobactam (TZP)	484 (95.5)	23 (4.5)	423 (95.9)	18 (4.1)	0.731
Trimethoprim/Sulfamethoxazole (SXT)	291 (70.8)	120 (29.2)	235 (62.5)	141 (37.5)	0.013
Tobramycin (TOB)	361 (76.5)	111 (23.5)	85 (63.4)	49 (36.6)	0.002

**Table 3:** Comparison of demographic data and risk factors among patients with community-acquired acute pyelonephritis

	2010–2011 (*n* = 818)	2017–2018 (*n* = 617)	*p*
Demographic Data			
Age (years), mean ± SD	56.66 ± 19.06	60.21 ± 18.73	<0.001
Female sex (%)	761 (93.0)	566 (91.7)	0.356
Underlying Comorbidities (%)			
Charlson’s comorbidity index, mean ± SD	0.81 ± 1.37	1.04 ± 1.40	0.002
Diabetes mellitus	216 (26.4)	196 (31.8)	0.026
Hemiplegia	19 (2.3)	10 (1.6)	0.349
Cerebrovascular accident	77 (9.4)	58 (9.4)	0.993
Congestive heart failure	44 (5.4)	19 (3.1)	0.035
Connective tissue disease	13 (1.6)	24 (3.9)	0.006
Malignancy	32 (3.9)	51 (8.3)	<0.001
Chronic pulmonary disease	21 (2.6)	23 (3.7)	0.207
Liver disease	26 (3.2)	36 (5.8)	0.014
Renal disease	46 (5.6)	49 (7.9)	0.084
Dementia	32 (3.9)	38 (6.2)	0.050
Pregnancy among female patients	8 (1.0)	4 (0.6)	0.497
Menopause among female patients	344/761 (45.2)	319/566 (56.4)	<0.001
Bedridden state	37 (4.5)	28 (4.5)	0.989
Underlying Urinary Tract Conditions (%)			
Indwelling urinary catheter	12 (1.5)	22 (3.6)	0.010
Intermittent catheterization	7 (0.9)	2 (0.3)	0.315
Benign prostatic hyperplasia among male patients	12/57 (21.0)	22/51 (43.1)	0.011
Neurogenic bladder	7 (0.9)	25 (4.1)	<0.001
Urolithiasis	15 (1.8)	22 (3.6)	0.040
Urinary retention	4 (0.5)	10 (1.6)	0.031
Vesicoureteral reflux	3 (0.4)	1 (0.2)	0.639
Vaginal wall prolapse among female patients	3/761 (0.4)	3/566 (0.5)	0.705
Polycystic kidney	6 (0.7)	2 (0.3)	0.478
Renal tumor	1 (0.1)	4 (0.6)	0.172
Past History (%)			
History of admission during 1 year prior to inclusion	188 (23.0)	164 (26.6)	0.117
History of antibiotic usage during 1 year prior to inclusion	227 (27.8)	191 (31.0)	0.179
History of urinary tract infection	209 (25.6)	145 (23.5)	0.373
Use of chemotherapeutic agents	13 (1.6)	10 (1.6)	0.962
Use of immunosuppressants	8 (1.0)	10 (1.6)	0.279
History of urinary catheterization during 1 month prior to inclusion	13 (1.6)	12 (1.9)	0.685
History of urinary tract operation during 3 months prior to inclusion	4 (0.5)	2 (0.3)	0.705

Abbreviations: SD, standard deviation.

**Table 4:** Comparison of clinical characteristics of patients with community-acquired acute pyelonephritis.

	2010–2011 (*n* = 818)	2017–2018 (*n* = 617)	*p*
Clinical Features			
Pitt’s score, mean ± SD	0.63 ± 1.07	0.69 ± 0.96	0.051
Urinary tract infection symptoms (%)	526 (64.3)	329 (53.3)	<0.001
Costovertebral angle tenderness (%)	522 (63.8)	409 (66.3)	0.331
Back pain (%)	260 (31.8)	170 (27.6)	0.083
Vomiting/diarrhea (%)	224 (27.4)	157 (25.4)	0.410
Hematuria (%)	436 (53.3)	307 (49.8)	0.183
Azotemia (%)	135 (16.5)	179 (29.0)	<0.001
Bacteremia (%)	262 (32.0)	269 (43.6)	<0.001
Antimicrobial Therapy			
Initial antimicrobial regimen			
Discordant to the antimicrobial susceptibility of causative organisms (%)	67/597 (11.2)	134/501 (26.8)	<0.001
ESC (%)	406 (49.6)	388 (62.9)	<0.001
FQ (%)	211 (25.8)	121 (19.6)	0.006
BL/BLI (%)	17 (2.1)	58 (9.4)	<0.001
Carbapenem (%)	13 (1.6)	38 (6.1)	<0.001
Duration of antibiotic therapy, days			
Total antibiotics, mean ± SD	16.55 ± 9.68	19.12 ± 9.90	<0.001
Parenteral antibiotics, mean ± SD	8.16 ± 6.44	10.17 ± 7.31	<0.001
ESC, mean ± SD	6.31 ± 8.13	7.82 ± 8.09	0.001
FQ, mean ± SD	4.96 ± 8.64	6.61 ± 9.58	0.001
BL/BLI, mean ± SD	0.94 ± 3.43	2.35 ± 5.13	<0.001
Carbapenem	0.59 ± 2.87	1.79 ± 4.89	<0.001
Days of change of antibiotics, days, median (IQR)	6 (4–8)	6 (4–7)	0.477
Outcomes			
Mortality (%)	11/734 (1.9)	2/597 (0.3)	0.003
Clinical failure (%)	39/734 (5.3)	13/597 (2.2)	0.003
Hospitalization days, median (IQR)	7 (6–10)	9 (7–13)	<0.001
Febrile days, median (IQR)	2 (1–3)	2 (1–4)	<0.001

Abbreviations: SD, standard deviation; ESC, extended-spectrum cephalosporins; FQ, fluoroquinolone; BL/BLI, beta-lactam/beta-lactamase inhibitor; IQR, interquartile range.

**Table 5:** Comparison of clinical and microbiological characteristics of patients with community-acquired acute pyelonephritis after propensity-score matching.

	2010–2011 (*n* = 617)	2017–2018 (*n* = 617)	*p*
Age (years), mean ± SD	60.45 ± 18.62	60.22 ± 18.73	0.827
Charlson’s comorbidity index, mean ± SD	0.97 ± 1.36	1.04 ± 1.40	0.398
Antibiotic Resistance Rate of Uropathogenic *E. coli* Isolates (%)			
Amikacin (AMK)	6/400 (1.5)	1/441 (0.2)	0.058
Aztreonam (ATM)	35/361 (9.7)	137/441 (31.1)	<0.001
Cefazolin (CFZ)	26/204 (12.7)	136/373 (36.5)	<0.001
Cefepime (FEP)	35/394 (8.9)	140/441 (31.7)	<0.001
Cefotaxime (CTX)	35/391 (9.0)	148/440 (33.6)	<0.001
Cefoxitin (FOX)	11/280 (3.9)	32/441 (7.3)	0.066
Fluoroquinolone (FQ)	79/396 (19.9)	141/441 (32.0)	<0.001
Gentamicin (GEN)	88/398 (22.1)	145/441 (32.9)	0.001
Piperacillin/tazobactam (TZP)	15/381 (3.9)	18/441 (4.1)	0.916
Trimethoprim/sulfamethoxazole (SXT)	87/317 (27.4)	141/376 (37.5)	0.005
Discordance of initial antibiotic regimen to the antimicrobial susceptibility of causative organisms (%)	61/461 (13.2)	134/501 (26.8)	<0.001
Total duration of antibiotic therapy, days, mean ± SD	16.94 ± 10.51	19.12 ± 9.90	<0.001
**Outcomes**			
Mortality (%)	11/549 (2.0)	2/597 (0.3)	0.008
Clinical failure (%)	33/549 (6.0)	13/597 (2.2)	0.001
Hospitalization days, median (IQR)	8 (6–11)	9 (7–13)	<0.001
Febrile days, median (IQR)	2 (1–3)	2 (1–4)	0.003

Abbreviations: SD, standard deviation; FQ, fluoroquinolone; IQR, interquartile range.

## Supplementary Materials

The following are available online at https://www.mdpi.com/2079-6382/9/9/617/s1, Table S1: Comparison of the aetiologies of community-acquired acute pyelonephriti (from the four hospitals, which participated in both periods), Table S2: Comparison of antibiotic susceptibilities of uropathogenic *E. coli* isolates (from the four hospitals, which participated in both periods), Table S3: Comparison of demographic data and risk factors among patients with community-acquired acute pyelonephritis (from the four hospitals, which participated in both periods), Table S4: Comparison of clinical characteristics of patients with community-acquired acute pyelonephritis (from the four hospitals, which participated in both periods).
