# Supplementary material for: Changes in Clinical Characteristics of Community-Acquired Acute Pyelonephritis and Antimicrobial Resistance of Uropathogenic Escherichia coli in South Korea in the Past Decade
Source: Antibiotics (Basel). 2020 Sep 18;9(9):617. doi: 10.3390/antibiotics9090617 (PMC7559700; doi:10.3390/antibiotics9090617)
Supplement: Supplementary file 1 [file antibiotics-09-00617-s001.pdf]

Table S1. Comparison of the aetiologies of community-acquired acute pyelonephriti (from the four hospitals, which participated in both periods)

Pathogens	2010-2011	2017-2018	*P*
Escherichia coli	291 (86.6)	260 (87.5)	0.727
Klebsiella pneumoniae	11 (3.3)	15 (5.1)	0.261
Proteus spp.	7 (2.1)	5 (1.7)	0.731
Enterobacter spp.	2 (0.6)	3 (1.0)	0.670
Citrobacter spp.	0 (0)	2 (0.7)	0.220
**Subtotal**	**311 (92.5)**	**285 (95.9)**	**0.069**
Pseudomonas aeruginosa	1 (0.3)	4 (1.3)	0.192
Acinetobacter baumanii	2 (0.6)	0 (0)	0.501
Enterococcus spp.	7 (2.1)	4 (1.3)	0.228
Staphylococcus aureus	2 (0.6)	0 (0)	0.501
Others	13 (3.9)	4 (1.3)	0.050
Total	336 (100)	297 (100)	-

Table S2. Comparison of antibiotic susceptibilities of uropathogenic *E. coli* isolates (from the four hospitals, which participated in both periods)

	2010-2011 (n=291)		2017-2018 (n=248)		
	Sensitive (%)	Resistant (%)	Sensitive (%)	Resistant (%)	*P*
Amikacin (AMK)	288 (99.0)	3 (1.0)	248 (100)	0 (0)	0.253
Amoxicillin/clavulanate (AMC)	152 (80.9)	36 (19.1)	155 (72.4)	59 (27.6)	0.047
Ampicillin (AMP)	101 (35.6)	183 (64.4)	61 (24.6)	187 (75.4)	0.006
Ampicillin/sulbactam (SAM)	55 (51.9)	51 (48.1)	39 (38.2)	63 (61.8)	0.048
Aztreonam (ATM)	260 (91.2)	25 (8.8)	172 (69.4)	76 (30.6)	<0.001
Cefazolin (CFZ)	120 (82.2)	26 (17.8)	111 (61.7)	69 (38.3)	<0.001
Cefepime (FEP)	262 (92.3)	22 (7.7)	169 (68.1)	79 (31.9)	<0.001
Cefotaxime (CTX)	269 (92.4)	22 (7.6)	161 (65.2)	86 (34.8)	<0.001
Cefoxitin (FOX)	174 (96.1)	7 (3.9)	231 (93.1)	17 (6.9)	0.184
Ceftazidime (CAZ)	267 (92.1)	23 (7.9)	171 (69.0)	77 (31.0)	<0.001
Fluoroquinolone (FQ)	229 (79.0)	61 (21.0)	165 (66.5)	83 (33.5)	0.001
Gentamicin (GEN)	231 (79.4)	60 (20.6)	166 (66.9)	82 (33.1)	0.001
Imipenem (IPM)	289 (99.7)	1 (0.3)	248 (100)	0 (0)	1.000
Meropenem (MEM)	218 (99.5)	1 (0.5)	102 (100)	0 (0)	1.000
Piperacillin (PIP)	69 (35.0)	128 (65.0)	21 (30.9)	47 (69.1)	0.534
Piperacillin/tazobactam (TZP)	274 (95.1)	14 (4.9)	234 (94.4)	14 (5.6)	0.684
Trimethoprim/sulfamethoxazole (SXT)	184 (71.6)	73 (28.4)	114 (62.3)	69 (37.7)	0.040
Tobramycin (TOB)	230 (79.3)	60 (20.7)	62 (61.4)	39 (38.6)	<0.001

Table S3. Comparison of demographic data and risk factors among patients with community-acquired acute pyelonephritis (from the four hospitals, which participated in both periods)

	2010-2011 (n=472)	2017-2018 (n=349)	P
Demographic data			
Age (years), mean ± SD	55.77±18.60	60.71±17.29	<0.001
Female sex (%)	441 (93.4)	317 (90.8)	0.166
Underlying co-morbidities (%)			
Charlson's comorbidity index, mean ± SD	0.68±1.17	1.04±1.39	<0.001
Diabetes mellitus	119 (25.2)	113 (32.4)	0.024
Hemiplegia	9 (1.9)	3 (0.9)	0.216
Cerebrovascular accident	36 (7.6)	34 (9.7)	0.283
Congestive heart failure	25 (5.3)	12 (3.4)	0.205
Connective tissue disease	8 (1.7)	22 (6.3)	0.001
Malignancy	15 (3.2)	32 (9.2)	<0.001
Chronic pulmonary disease	9 (1.9)	11 (3.2)	0.253
Liver disease	11 (2.3)	19 (5.4)	0.019
Renal disease	17 (3.6)	25 (7.2)	0.025
Dementia	15 (3.2)	16 (4.6)	0.296
Pregnancy among female patients	0 (0)	1 (0.3)	0.425
Menopause among female patients	256/441 (58.0)	147/326 (45.1)	<0.001
Bedridden state	14 (3.0)	13 (3.7)	0.547
Underlying urinary tract conditions (%)			
Indwelling urinary catheter	7 (1.5)	8 (2.3)	0.392
Intermittent catheterization	3 (0.6)	0 (0)	0.266
Benign prostatic hyperplasia	4/31 (12.9)	13/32 (40.6)	0.013
Neurogenic bladder	2 (0.4)	14 (4.0)	<0.001
Urolithiasis	9 (1.9)	6 (1.7)	0.843
Urinary retention	0 (0)	3 (0.9)	0.076
Vesicoureteral reflux	1 (0.2)	0 (0)	1.000
Vaginal wall prolapse	1/441 (0.2)	2/326 (0.6)	0.578
Polycystic kidney	4 (0.8)	0 (0)	0.141

Renal tumour	0 (0)	3 (0.9)	0.076
Past history (%)			
History of admission during 1 year prior to inclusion	104 (22.0)	88 (25.2)	0.287
History of antibiotic usage during 1 year prior to inclusion	115 (24.4)	108 (31.0)	0.034
History of urinary tract infection	106 (22.5)	72 (20.6)	0.530
Use of chemotherapeutic agents	7 (1.5)	6 (1.7)	0.789
Use of immunosuppressants	4 (0.8)	10 (2.9)	0.027
History of urinary catheterization during 1 month prior to inclusion	6 (1.3)	1 (0.3)	0.249
History of urinary tract operation during 3 months prior to inclusion	2 (0.4)	1 (0.3)	1.000

Abbreviations: SD, standard deviation

Table S4. Comparison of clinical characteristics of patients with community-acquired acute pyelonephritis (from the four hospitals, which participated in both periods)

	2010-2011 (n=472)	2017-2018 (n=349)	*P*
Clinical characteristics			
Pitt's score, mean ± SD	0.48±0.99	0.67±0.95	0.008
Urinary tract infection symptoms (%)	318 (67.4)	180 (51.6)	<0.001
Costovertebral angle tenderness (%)	305 (64.6)	230 (65.9)	0.703
Back pain (%)	147 (31.1)	53 (15.2)	<0.001
Vomiting/diarrhoea (%)	124 (26.3)	84 (24.1)	0.473
Haematuria (%)	245 (51.9)	166 (47.6)	0.219
Azotaemia (%)	77 (16.3)	98 (28.1)	<0.001
Bacteraemia (%)	154 (32.6)	137 (39.3)	0.050
Initial antibiotic regimen			
Discordant to the antimicrobial susceptibility of causative organisms (%)	303/332 (91.3)	206/282 (73.0)	<0.001
ESC (%)	248 (52.5)	271 (77.7)	<0.001
FQ (%)	22 (4.7)	9 (2.6)	0.122
BL/BLI (%)	12 (2.5)	46 (13.2)	<0.001
Carbapenem (%)	7 (1.5)	25 (7.2)	<0.001
Duration of total antibiotics, days, mean ± SD	15.40±6.35	21.74±11.72	<0.001
Days of change of antibiotics, days, median (IQR)	6 (4-8)	2 (2-6)	<0.001
Outcomes			
Mortality	11/452 (2.4)	1/342 (0.3)	0.014
Clinical failure (%)	17/452 (3.8)	7/342 (2.0)	0.162
Hospitalization days, median (IQR)	8 (6-11)	10 (7-14)	<0.001
Febrile days, median (IQR)	2 (1-3)	3 (1-4)	<0.001

Abbreviations: SD, standard deviation; ESC, extended-spectrum cephalosporins; FQ, fluoroquinolone; BL/BLI, beta-lactam/beta-lactamase inhibitor; IQR, interquartile range
